# Quality-of-Life Scores for Nursing Home Residents Are Stable Over Time: Evidence From Minnesota

**DOI:** 10.1093/geroni/igab046.273

**Published:** 2021-12-17

**Authors:** Weiwen Ng, Tetyana Shippee, John Bowblis, Odichinma Akosionu, Mark Woodhouse, Yinfei Duan

**Affiliations:** 1 University of Minnesota, Minneapolis, Minnesota, United States; 2 University of Minnesota, University of Minnesota, Minnesota, United States; 3 Miami University, Oxford, Ohio, United States

## Abstract

**Objective:**

Quality of life (QoL) is a multidimensional construct that assesses the quality of lived experience in nursing homes (NHs). QoL is directly important to NH residents. However, QoL is only publicly reported in a few states, partly because of concerns regarding measure stability. To address these concerns, we tested the stability of Minnesota’s NH QoL measure over one year.

**Study Design:**

A pair of two-year cohorts of Minnesota NH residents who responded to the 2012-2013 (N = 4,448) or 2014-2015 (N = 4,644) QoL survey in consecutive years. Stability was measured using the intra-class correlation (ICC) from hierarchical linear models. Models were fit without any covariates, then individual and facility-level characteristics were added. Principal Findings: Overall QoL had ICCs of 0.602 and 0.614 in the earlier and later cohort respectively. Domain-level ICCs were lower, ranging from 0.374 (positive mood) to 0.571 (lack of negative mood) in the 2012-2013 cohort, with similar trends for the later cohort. Adjusting for important covariates reduces the ICCs slightly, but they remained at 0.565 or higher for the summary score.

**Conclusions:**

Person-reported summary QoL has adequate stability over a period of one year. Our results provide impetus to assess and report NH QoL on a national level. Consumers can be confident that if an NH’s QoL scores improve from year to year, that represents a real improvement, and not just the scores varying due to which residents were sampled. Some caution, however, is warranted when presenting facility-level domain scores, as these are less stable.

